# Editorial: Highlights in pediatric pain 2021/22

**DOI:** 10.3389/fpain.2023.1152194

**Published:** 2023-03-17

**Authors:** Tonia C. Onyeka, Rocio de la Vega, Emma Fisher, G. Allen Finley

**Affiliations:** ^1^ Department of Anaesthesia/Pain & Palliative Care Unit, College of Medicine, University of Nigeria, Ituku-Ozalla, Enugu, Nigeria; ^2^ Center for Translation and Implementation Research, University of Nigeria, Nsukka, Nigeria; ^3^ Faculty of Psychology, University of Malaga, Malaga, Spain; ^4^ Biomedical Research Institute of Málaga (IBIMA), Malaga, Spain; ^5^ Centre for Pain Research, University of Bath, Bath, United Kingdom; ^6^ Department of Anesthesia, Pain Management & Perioperative Medicine, Dalhousie University, Halifax, NS, Canada

**Keywords:** pediatric pain, pain mechanisms, pain treatment, biomarkers, risk factors, cognitive behavioral therapy (CBT), COVID-19

**Editorial on the Research Topic** Highlights in pediatric pain 2021/22

Chronic pain affects one in four children and adolescents (1–3) and has been declared a global health priority (4) because of its economic burden to society (5) and substantial impact on child and family (6). Psychological treatments have been found to be effective in reducing pain intensity and disability (7, 8), however, there is considerable variability in treatment response, such that some children and adolescents benefit while others do not and more research has been called for in this area. This editorial highlights the broad diversity of research performed across the Pediatric Pain section, including a summary of pediatric chronic pain management modalities, from bench to bedside, and an intersect of the biopsychosocial aspects of pain. It includes five manuscripts: three original studies, one brief research report and one mini review.

The review article by Jotwani et al. highlights the importance of behavioural treatments in pediatric chronic pain, the latter being widespread and of complex nature as a result of the progressive growth of the nervous system (9). The authors found mixed efficacy of psychological interventions for pain outcomes, proposing interactions between psychosocial factors such as pain catastrophizing and biobehavioral mechanisms like inflammatory biomarkers, and the relevance of such interactions to patient treatment outcomes, the authors posit that in order to further expand this vital research area, conducting mechanistic clinical trials (which are studies involving human participants to have a better understanding of biologic or behavioral mechanisms) (10) utilizing mixed methods may help explain the mixed results. They suggest that neuroimaging techniques (especially fNIRs imaging) to observe for neurologic pain processes and artificial intelligence to detect pain predictors are new frontiers in mechanistic pediatric pain research.

In line with the suggestions of the review by Jotwani et al. Lalouni et al. explored the role of parental factors on psychological treatment effects. They conducted a secondary data from a study that randomized 90 children (8- to 12-year-olds) to receive either exposure-based online cognitive behavioral therapy (ICBT) or usual care, to look at the influence of parental protective behaviours (such as allowing child stay at home on a school day), parental catastrophizing and monitoring behaviours on children with functional abdominal pain disorder (FAPD). Parental catastrophizing only was associated less change in pediatric quality of life after treatment. Findings from this study encourage parent-child co-treatment and the necessity of parental catastrophizing treatments for the benefit of children with FAPD.

The research by Semkovych and Dmytriiev focuses on an under-researched area: the use of biomarkers to track pain progression in clinical practice (11). They investigate the role of biomarkers of the innate immune system to address this issue. Their 3-arm randomized controlled trial (RCT) conducted in children who underwent inguinal herniotomy, examined the relationship between postoperative Toll-like receptor 4 (TLR4) levels, a pro-inflammatory biomarker implicated in the development of neuropathic pain, different perioperative analgesic regimens and their effect on pain chronification. Acute pain was found to be significantly higher in the general anesthesia (GA) group than the Quadratus Lumborum Block (QLB) group and the Transversalis Fascia Plane Block (TFPB) group. Chronic pain was found to be prevalent in the GA group when compared with the QLB and TFPB groups. They conclude by summarizing the benefits of regional anaesthesia for children to include ease of use, adequate perioperative pain relief, reduction in opioid requirements and reduced TLR4 levels, reducing the possibility of pain chronicity.

Regarding risk factors for paediatric pain, the brief research report by Borschneck et al. sheds some light on risk factors for anterior knee pain (AKP) in adolescents, a condition considered to be responsible for up to 40% of visits to the physiotherapist (12). They elucidate the intrinsic and extrinsic risk factors that have been found to increase the risk of AKP including participation in sports but highlight the observation that there is a lack of information regarding the association between participation in specific sports and increased risk of AKP. They found that previous AKP and previously treated knee injuries seem to be risk factors especially in females. In addition, participation in basketball, baseball, cross-country, riding, and volleyball, are associated with increases the risk of AKP.

Finally, Kaczynskyi et al. conducted a peri-pandemic assessment of pain and other psychosocial outcomes among children and adolescents diagnosed with chronic pain disorders and their families. In comparing functioning of participants during two periods and in two situations: outside the home pre-pandemic and inside the home during the COVID-19 pandemic, the authors found significant decreases in pain intensity, pain catastrophizing (including rumination and helplessness) and significant decreases in psychological stress and anxiety during the first few months of the lockdown. The authors surmise that although reduction in pain was experienced when participants were removed from the traditional school setting, teaching children effective coping strategies and self-advocacy skills will help them adapt to any environment they find themselves in.

## Conclusions

The articles composing this collection provide key insights for pediatric pain assessment and psychological treatment and a path forward to advance the field, focusing on identifying biomarkers, moderators and mediators of treatment efficacy and using mixed methods to better-understand mechanisms of change. They identify the need to take a more directed approach to many aspects of pediatric pain care, and to seek an understanding of specific biological and cognitive mechanisms in the diagnosis, assessment, and management of pain in children. We look forward to other innovative and exciting submissions on pediatric pain research.
